# Role of Responsive Neurostimulation in Managing Drug-Resistant Epilepsy: A Systematic Review of Clinical Outcomes

**DOI:** 10.7759/cureus.68032

**Published:** 2024-08-28

**Authors:** Omid Mushtaq, Han Grezenko, Abdur Rehman, Hamza Sher, Zarrar Sher, Delphyne Anyang Kaakyire, Syed Hanifullah, Muath Dabas, Ghaida Saleh, Abdullah Shehryar, Isa Khan

**Affiliations:** 1 Diagnostic and Interventional Radiology, Sakarya University Faculty of Medicine, Sakarya, TUR; 2 Medicine and Surgery, Guangxi Medical University, Nanning, CHN; 3 Translational Neuroscience, Barrow Neurological Institute, Phoenix, USA; 4 Surgery, Mayo Hospital, Lahore, PAK; 5 Internal Medicine, Islamic International Medical College, Rawalpindi, PAK; 6 Internal Medicine, Tehsil Headquarter Hospital (THQ) Hospital Kotli Sattian, Rawalpindi, PAK; 7 Internal Medicine, Saint Petersburg State University, Saint Petersburg, USA; 8 Neurosurgery, Prime Hospital Complex, Peshawar, PAK; 9 Surgery, University of Jordan, Amman, JOR; 10 Internal Medicine, University of Khartoum, Khartoum, SDN; 11 Internal Medicine, Allama Iqbal Medical College, Lahore, PAK; 12 Internal Medicine, Nishtar Medical University, Multan, PAK

**Keywords:** systematic review, quality of life, seizure reduction, drug-resistant epilepsy, responsive neurostimulation

## Abstract

Drug-resistant epilepsy remains a substantial challenge in neurology, affecting patients who do not respond to conventional antiepileptic drugs. Responsive neurostimulation (RNS) has emerged as a promising therapeutic approach, yet comprehensive reviews synthesizing its clinical outcomes are sparse. This systematic review followed Preferred Reporting Items for Systematic Reviews and Meta-Analyses (PRISMA) guidelines and involved a comprehensive database search through PubMed, Medline, Embase, the Cochrane Library, and Scopus, covering literature up to April 2024. The review targeted peer-reviewed articles evaluating the efficacy, safety, and quality of life impacts of RNS in patients with drug-resistant epilepsy. Key inclusion criteria encompassed clinical trials, cohort studies, and meta-analyses, while exclusion criteria included non-peer-reviewed and irrelevant studies. We identified five studies meeting our inclusion criteria. These studies collectively demonstrated that RNS significantly reduces seizure frequency and improves quality of life, while maintaining a favorable safety profile. Despite small sample sizes and potential selection biases, the benefits of RNS appeared consistent across diverse patient demographics. RNS represents a viable and effective treatment option for drug-resistant epilepsy, offering significant improvements in seizure control and patient quality of life. Future research should focus on long-term outcomes and refining patient selection to optimize the therapeutic benefits of RNS. The integration of RNS into standard epilepsy management protocols is recommended based on current evidence.

## Introduction and background

Responsive neurostimulation (RNS) represents a significant advancement in the management of drug-resistant epilepsy, a condition characterized by persistent seizures despite treatment with multiple antiepileptic drugs [1]. Traditional management strategies often fall short for a substantial subset of patients, making innovative treatments essential. RNS operates on a closed-loop system, detecting abnormal electrical activity in the brain and delivering electrical pulses to normalize it before symptoms of a seizure begin [2]. This approach not only holds promise for reducing seizure frequency but also aims at improving overall quality of life while minimizing adverse effects associated with other treatments such as resective surgery or chronic medication [3].

Recent technological advancements and increased clinical application of RNS have generated a plethora of data on its efficacy and safety [4]. However, the rapidly evolving nature of this field and the diversity in patient outcomes call for a comprehensive synthesis of available evidence. This is crucial not only for elucidating the effectiveness and safety of RNS but also for understanding its potential limitations and areas requiring further investigation [5].

The primary objective of this systematic review is to critically assess and synthesize the clinical outcomes associated with RNS in patients with drug-resistant epilepsy. Specifically, the review aims to evaluate the impact of RNS on seizure reduction, quality of life, and the prevalence of adverse effects. By integrating findings from recent studies, this review seeks to offer insights into the effectiveness of RNS as a treatment option, identify patterns in responsiveness, and discuss the implications of these outcomes for future clinical practice and research. This comprehensive analysis will aid healthcare providers in making informed decisions and contribute to the optimization of treatment strategies for patients suffering from this challenging neurological disorder.

## Review

Methodology

Search Strategy

Our search strategy was rigorously designed in alignment with the Preferred Reporting Items for Systematic Reviews and Meta-Analyses (PRISMA) guidelines to explore the efficacy and safety of RNS in treating drug-resistant epilepsy. We executed detailed searches across several prominent electronic databases, including PubMed, Medline, Embase, the Cochrane Library, and Scopus. The search period covered from the inception of each database up to April 2024.

We utilized a combination of keywords and medical subject headings (MeSH) terms pertinent to our research question, such as “epilepsy,” “drug-resistant epilepsy,” “refractory epilepsy,” “neurostimulation,” “responsive neurostimulation,” and “RNS.” Boolean operators (AND, OR) were employed to integrate these terms efficiently. Example search strings used were “responsive neurostimulation AND drug-resistant epilepsy,” “refractory epilepsy AND RNS outcomes,” and “neurostimulation AND epilepsy treatment efficacy.”

To enhance the scope of our search and capture a broad spectrum of relevant studies, we also reviewed the reference lists of all selected articles for additional relevant literature. Our strategy further included searches in clinical trial registries and relevant neurological and neurosurgical conference proceedings to identify unpublished or ongoing studies that might provide additional insights into the current research landscape.

The search was confined to studies published in the English language and peer-reviewed journals. Our inclusion criteria were specifically devised to capture clinical trials, cohort studies, case series, and meta-analyses focusing on the clinical outcomes of RNS in individuals with drug-resistant epilepsy. This meticulous approach ensured the capture of the most relevant and up-to-date data, facilitating a comprehensive analysis of the topic.

Eligibility Criteria

The eligibility criteria for this systematic review were meticulously formulated to ensure the inclusion of studies that were scientifically robust and directly relevant to the management of drug-resistant epilepsy using RNS. We included peer-reviewed research articles such as clinical trials, randomized controlled trials (RCTs), observational studies, cohort studies, and meta-analyses. These studies needed to specifically investigate the efficacy and safety of RNS in patients diagnosed with drug-resistant epilepsy. Relevant outcomes included seizure frequency reduction, responder rates, quality of life improvements, and safety profiles. All considered studies needed to be published in the English language and ranged from the inception of the respective databases to April 2024 to ensure that the review encompassed the most up-to-date research.

Conversely, our exclusion criteria were designed to omit studies that did not align with the review’s core objectives. Studies that did not focus on RNS or its direct impacts on patients with drug-resistant epilepsy were excluded. We also excluded non-peer-reviewed articles, grey literature such as conference abstracts and unpublished works, and studies published in languages other than English to avoid the complexities of translation that might affect the interpretation of data. Additionally, studies that lacked comprehensive outcome data or clear methodological descriptions that prevent a thorough assessment of the interventions’ effects were also excluded. This strategic selection process ensured that our review maintained a high standard of relevance and scientific integrity, providing clear insights into the effectiveness and safety of RNS in this challenging clinical context.

Data Extraction

The data extraction process for our systematic review of RNS as a treatment for drug-resistant epilepsy was rigorously structured to ensure both the reliability and thoroughness of the data collected. Initially, all articles were screened based on their titles and abstracts. Two independent reviewers assessed these articles to determine their relevance to our study objectives, categorizing them as “relevant,” “not relevant,” or “potentially relevant.” This initial screening was vital to focus our review on the most pertinent studies.

Subsequently, articles identified as potentially relevant underwent a detailed full-text review. To standardize the data collection process across all studies, we utilized a custom-designed form in Microsoft Excel. Each reviewer independently filled out this form, applying our pre-established inclusion and exclusion criteria to ensure that only the most applicable studies were considered. In cases of disagreement or ambiguity regarding an article’s eligibility, a third reviewer was consulted to resolve the discrepancy through discussion, ensuring a consensus-based approach and enhancing the accuracy of our data collection.

Our data extraction form was tailored to collect key information essential for addressing the review’s aims. This included the lead author’s name, publication year, study type, sample size, primary outcomes regarding the efficacy and safety of RNS, secondary outcomes, and any noted study limitations or biases. This methodical approach facilitated a thorough and systematic evaluation of each selected study, allowing for a comprehensive synthesis of the evidence necessary to assess the effectiveness and safety of RNS in managing drug-resistant epilepsy.

Data Analysis and Synthesis

Due to the variability in study designs and patient demographics, we opted for a qualitative synthesis over a meta-analysis for our review of RNS in drug-resistant epilepsy. This approach allowed for a comprehensive exploration of RNS outcomes, integrating findings across different studies to identify common themes and significant differences in efficacy, safety, and patient quality of life.

We categorized key findings to discern patterns related to seizure reduction, responder rates, and adverse events, providing a detailed overview of RNS effectiveness. Our narrative synthesis offered insights into current research trends, highlighted gaps, and suggested future directions for optimizing RNS treatment strategies. This qualitative approach enabled a robust evaluation of the evidence, contributing valuable insights into RNS as a treatment option for drug-resistant epilepsy.

Results

Study Selection Process

**Figure 1:**
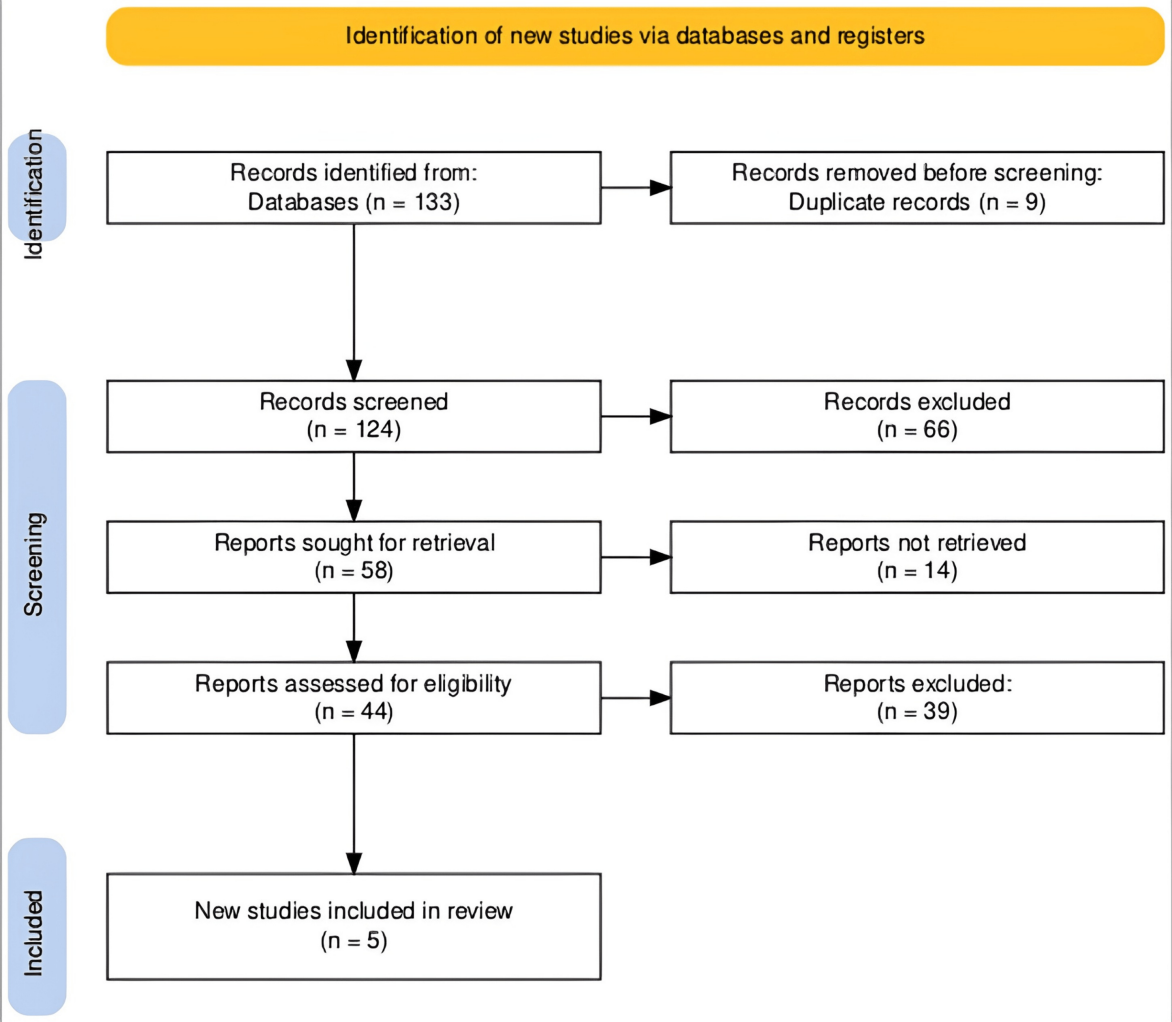
The PRISMA flowchart of study selection for a systematic review on responsive neurostimulation in drug-resistant epilepsy. PRISMA: Preferred Reporting Items for Systematic Reviews and Meta-Analyses

The search across various databases yielded a total of 133 records. After the removal of nine duplicates, 124 records were screened for relevance. Of these, 58 records were selected for more detailed evaluation. Following a thorough assessment for eligibility based on the predefined criteria, 44 reports were examined closely, resulting in the inclusion of five new studies in the systematic review. This methodical process is visually represented in the PRISMA flowchart (Figure 1), ensuring transparency and replicability in the selection of studies.

Characteristics of Selected Studies

Our systematic review incorporated five studies, each contributing unique insights into the efficacy and safety of RNS in treating drug-resistant epilepsy. These included a mix of RCTs, observational studies, and meta-analyses conducted between 2015 and 2023. Key studies offered comprehensive data on seizure reduction rates and safety profiles, demonstrating the significant impact of RNS on reducing seizure frequency and enhancing patient quality of life. Other studies focused on specific patient demographics, such as pediatric populations, and explored long-term outcomes and complications associated with RNS. Common limitations noted across studies were small sample sizes, short follow-up periods, and potential biases in study selection. This information is succinctly summarized in Table 1.

**Table 1:** Summary of selected studies evaluating RNS in drug-resistant epilepsy. VNS = vagus nerve stimulation; RNS = responsive neurostimulation; DBS = deep brain stimulation; PRISMA = Preferred Reporting Items for Systematic Reviews and Meta-Analyses; RCTs = randomized controlled trials; DRE = drug-resistant epilepsy; CI = confidence interval; IPDMA = individual participant data meta-analysis; QOL = quality of life; IQR = interquartile range

Authors and year	Objective	Methods	Primary outcome	Results - seizure reduction	Results - other	Complications	Significance
Touma et al., 2022 [6]	Summarize efficacy and tolerability of VNS, RNS, and DBS	Systematic review, meta-analysis, PRISMA standards, RCTs and case series with ≥20 adults	Mean or median percentage decrease in seizure frequency	VNS: ~34.7%; RNS: 53%, 66%, and 75% at 2, 5, and 9 years, respectively; DBS: 56%, 65%, and 75% at 2, 5, and 7 years, respectively	Increased seizure freedom over time for DBS and RNS	VNS: hoarseness, cough, and throat pain; RNS/DBS: implant-site pain, headache, and dysesthesia	Effective treatment with a few major complications, improving outcomes over time
Kusyk et al., 2022 [7]	Evaluate the efficacy and safety of RNS in DRE	Systematic review, meta-analysis, PRISMA protocols, 313 studies screened, 17 included, 541 patients	Mean seizure reduction rate, responder rate (>50% reduction)	Mean seizure reduction: 68% (95% CI: 61%-76%)	Mean responder rate: 68% (95% CI: 60%-75%)	18.9% complication rate, including device-related infections	Comparable efficacy to other implants, publication bias toward positive outcomes
Kerezoudis et al., 2022 [8]	Evaluate the safety of efficacy of RNS in pediatric epilepsy	Comprehensive literature search, review on pediatric epilepsy, 8 studies and 4 cases included	Reduction in seizure frequency at last follow-up vs. baseline	Median reduction: 75% (IQR: 50-88%)	80% responders; varied by epilepsy type	8% complication rate, four infections, no major deficits	Promising safety and efficacy profile; need for more rigorous trials
Levy et al., 2023 [9]	Elucidate effects of RNS in pediatric DRE	IPDMA, management of pediatric DRE via RNS, searched four databases, 15 studies, 98 participants	Median percent seizure reduction at follow-up	Median reduction: 75% at 12 months	57% achieved Engel Class <2; 9.7% seizure freedom	8.4% complication rate, half device-related infections	Effective treatment with a reasonable safety profile, highlights the need for further study
Meador et al., 2015 [10]	Assess the impact of RNS on QOL and mood in resistant focal epilepsy	RCT, double-blinded, 191 patients, followed up for 2 years, QOL and mood surveys	Changes in QOL and mood from baseline through the study	Improvement in QOL observed regardless of seizure origin	At 2 years, 44% reported QOL improvements, 16% declined; no adverse mood changes	Not specified	RNS associated with QOL improvements without adversely affecting mood

The quality of five key studies concerning neurostimulation for epilepsy was rigorously assessed using appropriate evaluation tools tailored to the study designs. For systematic reviews and meta-analyses, the Measurement Tool to Assess Systematic Reviews (AMSTAR 2) tool was employed, which evaluates aspects such as the comprehensiveness of the literature search and the adequacy of study selection. The Risk Of Bias In Non-randomized Studies-Interventions (ROBINS-I) tool was utilized for non-randomized studies, focusing on the risk of bias across multiple domains, including study confounding and selection of participants. Lastly, the Cochrane Collaboration’s Risk of Bias tool was applied to RCTs to scrutinize elements such as random sequence generation and blinding efficacy. Table 2 consolidates these assessments, summarizing the strengths and limitations of each study in key methodological domains, thereby providing a comparative overview of their rigor and reliability. A summary of quality assessment is presented in Table 2.

**Table 2:** Comprehensive quality assessment of five studies on neurostimulation. PICO = Population, Intervention, Comparator, Outcomes; AMSTAR 2 = A Measurement Tool to Assess Systematic Reviews, version 2; ROBINS-I = Risk Of Bias In Non-randomized Studies-Interventions; N/A = not applicable

Study	Assessment tool used	PICO criteria	Protocol registration	Explanation for included studies	Comprehensive literature search	Grey literature	Exclusion details	Included study details	Risk of bias assessment	Meta-analysis appropriateness	Bias interpretation	Publication bias	Conflicts of interest	Funding sources
Touma et al., 2022 (systematic review and meta-analysis)	AMSTAR 2	Yes	No	Yes	Yes	No	No	Partial	Yes	Yes	Yes	No	Yes	No
Kusyk et al., 2022 (systematic review and meta-analysis)	AMSTAR 2	Yes	No	Yes	Yes	No	Partial	Yes	Yes	Yes	Yes	Yes	No	No
Levy et al., 2023 (individual patient data Meta-analysis)	AMSTAR 2	Yes	Yes	Yes	Yes	No	Partial	Yes	Yes	Yes	Yes	No	No	No
Kerezoudis et al., 2022 (institutional review and meta-analysis)	ROBINS-I	Yes	No	Yes	Yes	No	Partial	Yes	Yes	Yes	Moderate	No	No	No
Meador et al., 2015 (randomized controlled trial)	Cochrane Collaboration’s Risk of Bias Tool	Yes	Yes	Yes	Yes	Unclear	Unclear	Yes	Yes	N/A	Low	Unclear	Unclear	Unclear

Discussion

Our systematic review of RNS in managing drug-resistant epilepsy has consolidated evidence from multiple studies, underscoring RNS as an effective treatment modality. Key findings across studies, such as those by Kusyk et al. [7] and Levy et al. [9], demonstrate a significant reduction in seizure frequency, with median reductions reaching up to 75% and substantial improvements in responder rates. Additionally, RNS has been shown to enhance the quality of life for many patients, with studies by Meador et al. [10] reporting that 44% of participants experienced meaningful quality of life improvements after RNS treatment. The safety profile of RNS was also favorable, with relatively low complication rates noted in pediatric and adult populations, suggesting that RNS is a viable and safe treatment option for those with drug-resistant epilepsy.

Our findings on the efficacy and safety of RNS align with and extend the current literature, offering deeper insights into its therapeutic potential for drug-resistant epilepsy [11]. Previous studies, such as those by Kusyk et al. [7], have documented the effectiveness of RNS in seizure reduction, which is corroborated by our systematic review showing consistent improvements across diverse patient groups. Notably, our review identified broader implications for quality of life improvements, a key aspect that enhances the clinical relevance of RNS beyond mere seizure control [12].

However, the degree of variability in seizure reduction outcomes and quality of life improvements noted in our review points to a potential influence of patient selection criteria, electrode placement, and stimulation parameters, which were not uniformly reported across all studies [13,14]. This variability underscores the necessity for standardized reporting practices in future RNS research to better understand the optimal conditions for efficacy. Additionally, our review suggests new correlations between patient age, epilepsy-onset location, and RNS effectiveness, particularly highlighting how younger patients and those with neocortical onset may experience different therapeutic outcomes [15]. These findings encourage a more tailored approach to RNS applications, potentially guiding more personalized treatment plans in clinical settings [16].

RNS is believed to exert its therapeutic effects through several neurophysiological mechanisms, primarily by detecting abnormal electrical activity and delivering targeted electrical stimulation to prevent seizure manifestation [17]. This “closed-loop” system likely modifies epileptic networks by interrupting seizure propagation and enhancing local inhibitory processes, which contributes to the observed reductions in seizure frequency [18]. Our review supports theories suggesting that RNS may induce long-term changes in neural plasticity, potentially reconfiguring neuronal networks toward a more stable and less excitable state. Such insights into the mechanisms of RNS contribute to a better understanding of epilepsy as a network disorder and open avenues for refining stimulation protocols to maximize therapeutic benefits [19].

The findings of our systematic review have significant implications for the clinical management of drug-resistant epilepsy. RNS can be integrated into current treatment protocols as a viable option for patients who do not respond to pharmacotherapy or are not suitable candidates for invasive surgery [20]. The effectiveness of RNS, as demonstrated in studies such as those by Kusyk et al. [7] and Levy et al. [9], highlights its potential to change standard care practices by providing a less invasive, customizable treatment alternative. However, successful integration of RNS requires careful consideration of patient selection, precise implantation strategies, and meticulous customization of stimulation parameters. These factors are critical in optimizing patient outcomes and underline the need for specialized training for clinical teams to implement and manage RNS therapy effectively [21].

The existing studies included in our systematic review, while informative, are not without limitations that may affect the generalizability of the findings. Common issues include small sample sizes and short follow-up periods that may not fully capture long-term outcomes and potential late complications of RNS treatment. Additionally, the potential for selection bias and lack of control groups in some studies could skew results favorably toward RNS. Despite these challenges, the strength of this systematic review lies in its comprehensive search strategy and rigorous analytical methods, which have enabled a thorough synthesis of available data across diverse study designs and patient populations. This approach enhances the reliability of our conclusions and provides a robust platform for understanding the impact of RNS on drug-resistant epilepsy.

The findings from this review underscore several avenues for future research that could further refine the therapeutic use of RNS in epilepsy management. There is a critical need for more RCTs that compare RNS directly with other neuromodulation therapies, such as vagus nerve stimulation or deep brain stimulation, to delineate their relative efficacies and safety profiles [22]. Additionally, long-term outcome studies are essential to assess the sustainability of seizure reduction and quality of life improvements over time and to identify any late-emerging side effects [23,24]. Research focusing on specific subgroups, such as pediatric patients or those with different epilepsy etiologies [25], would help tailor RNS treatments more effectively to individual patient needs and circumstances. These studies will provide deeper insights into the optimal use of RNS and potentially expand its indications within the field of neurology.

Our systematic review has revealed novel insights into the use of RNS in the treatment of drug-resistant epilepsy, particularly highlighting the importance of patient-specific factors in predicting treatment success. This personalized approach in applying RNS therapy, such as tailoring stimulation parameters and implantation strategies to individual patient characteristics and seizure profiles, represents an innovative shift toward more customized epilepsy care [26]. These findings suggest a potential for developing adaptive RNS systems that dynamically adjust to changes in patients’ seizure patterns over time, which could significantly enhance treatment efficacy and patient outcomes [27]. This personalized strategy opens new avenues for research and may lead to more sophisticated, artificial intelligence-driven neurostimulation therapies in the future [28].

The effectiveness and safety profile of RNS, as demonstrated in our review, have significant implications for healthcare policy and the management of epilepsy. The integration of RNS as a standard care option could necessitate updates to current epilepsy treatment guidelines, emphasizing the role of neuromodulation in comprehensive care plans [11]. Additionally, although upfront costs of RNS are considerable, the potential reduction in hospitalizations and emergency visits due to better seizure control could justify these expenses, leading to long-term healthcare savings. Policymakers might need to consider adjustments in healthcare coverage and reimbursement policies to facilitate broader access to RNS treatments, ensuring that more patients can benefit from this advanced therapeutic option without financial hardship [29].

Incorporating patient perspectives on RNS highlights critical aspects of patient satisfaction, adherence, and preference, which are essential for the broader adoption and success of RNS therapy [30]. Our review indicates that while many patients experience significant improvements in quality of life and seizure control, the invasiveness of the procedure and the device management required can affect patient compliance and satisfaction [31]. Understanding these personal experiences and preferences is crucial for clinicians to address concerns effectively and to tailor treatment plans that align with patients’ expectations and lifestyles. Enhanced patient education on the benefits and challenges of RNS, along with ongoing support and engagement, are key strategies that could improve patient acceptance and satisfaction, ultimately influencing the successful implementation and sustained use of RNS in clinical practice [32].

## Conclusions

Our systematic review comprehensively demonstrates that RNS offers a significant therapeutic benefit for patients suffering from drug-resistant epilepsy. The collected data across various studies confirm that RNS effectively reduces seizure frequency, improves quality of life, and maintains a favorable safety profile. These outcomes not only reinforce the role of RNS as a viable alternative to traditional treatments but also highlight its potential as a life-altering intervention for those who have exhausted other options. The review underlines the need for personalized treatment approaches, suggesting that future advancements should focus on optimizing RNS applications tailored to individual patient profiles. While challenges in patient selection, cost, and procedural considerations persist, the promising results from this review advocate for the broader inclusion of RNS in epilepsy treatment protocols, urging healthcare systems to adapt and provide greater access to this transformative technology. The integration of RNS into standard care practices could fundamentally change the management of epilepsy, offering renewed hope and improved outcomes for patients navigating this complex condition.
